# Attributable risk factors for asymptomatic malaria and anaemia and their association with cognitive and psychomotor functions in schoolchildren of north-eastern Tanzania

**DOI:** 10.1371/journal.pone.0268654

**Published:** 2022-05-26

**Authors:** Geofrey Makenga, Vito Baraka, Filbert Francis, Daniel T. R. Minja, Samwel Gesase, Edna Kyaruzi, George Mtove, Swabra Nakato, Rashid Madebe, Sif R. Søeborg, Kathrine H. Langhoff, Helle S. Hansson, Michael Alifrangis, John P. A. Lusingu, Jean-Pierre Van geertruyden

**Affiliations:** 1 National Institute for Medical Research, Tanga Centre, Tanga, Tanzania; 2 Global Health Institute, University of Antwerp, Antwerp, Belgium; 3 College of Education (DUCE), University of Dar es Salaam, Dar es Salaam, Tanzania; 4 Center for Medical Parasitology, Department of Immunology and Microbiology, University of Copenhagen, Copenhagen, Denmark; 5 Department of Infectious Diseases, Copenhagen University Hospital, Copenhagen, Denmark; Haramaya University, ETHIOPIA

## Abstract

In Africa, children aged 5 to 15 years (school age) comprises more than 50% (>339 million) of the under 19 years population, and are highly burdened by malaria and anaemia that impair cognitive development. For the prospects of improving health in African citizens, understanding malaria and its relation to anaemia in school-aged children, it is crucial to inform targeted interventions for malaria control and accelerate elimination efforts as part of improved school health policy. We conducted a study to determine the risk factors for asymptomatic malaria and their association to anaemia. We explored the prevalence of antimalarial drug resistance as well as the association of asymptomatic malaria infection and anaemia on cognitive and psychomotor functions in school-aged children living in high endemic areas. This study was a comprehensive baseline survey, within the scope of a randomised, controlled trial on the effectiveness and safety of antimalarial drugs in preventing malaria and its related morbidity in schoolchildren. We enrolled 1,587 schoolchildren from 7 primary schools located in Muheza, north-eastern Tanzania. Finger-pricked blood samples were collected for estimation of malaria parasitaemia using a microscope, haemoglobin concentration using a haemoglobinometer, and markers of drug resistance processed from dried blood spots (DBS). Psychomotor and Cognitive functions were assessed using a ‘20 metre Shuttle run’ and a test of everyday attention for children (TEA-Ch), respectively. The prevalence of asymptomatic malaria parasitaemia, anaemia and stunting was 26.4%, 49.8%, and 21.0%, respectively with marked variation across schools. In multivariate models, asymptomatic malaria parasitaemia attributed to 61% of anaemia with a respective population attribution fraction of 16%. Stunting, not sleeping under a bednet and illiterate parent or guardian were other factors attributing to 7%, 9%, and 5% of anaemia in the study population, respectively. Factors such as age group (10–15 years), not sleeping under a bednet, low socioeconomic status, parents’ or guardians’ with a low level of education, children overcrowding in a household, and fewer rooms in a household were significantly attributed to higher malaria infection. There was no significant association between malaria infection or anaemia and performance on tests of cognitive function (sustained attention) or psychomotor function (VO2 max). However, a history of malaria in the past one month was significantly associated with decreased cognitive scores (aOR = -4.1, 95% CI -7.7–0.6, *p = 0*.*02*). Furthermore, stunted children had significantly lower VO2max scores (aOR = -1.9, 95% CI -3.0–0.8, *p = 0*.*001*). Regarding the antimalarial drug resistance markers, the most prevalent *Pfmdr1* 86-184-1034-1042-1246 haplotypes were the NFSND in 47% (n = 88) and the NYSND in 52% (n = 98). The wild type *Pfcrt* haplotypes (codons 72–76, CVMNK) were found in 99.1% (n = 219) of the samples. Malaria, stunting and parents’ or guardians’ illiteracy were the key attributable factors for anaemia in schoolchildren. Given malaria infection in schoolchildren is mostly asymptomatic; an addition of interventional programmes such as intermittent preventive treatment of malaria in schoolchildren (IPTsc) would probably act as a potential solution while calling for an improvement in the current tools such as bednet use, school food programme, and community-based (customised) health education with an emphasis on nutrition and malaria control.

## Introduction

Africa is the second most populous continent in the world, with over 1.3 billion people (by the year 2020), of whom 52% are below 19 years. Children aged 5 to 15 years (schoolchildren) comprise of more than 50% (over 339 Million) of the under 19 years population [1,2]. For the prospect of building healthy citizens especially in Africa, focus on school health is therefore paramount. The World Health Organisation (WHO) 2019 malaria report estimated 228 million cases and 405,000 deaths related to malaria, while the African region bearing 93% of all cases [3]. In the sub Saharan African countries, malaria, schistosomiasis and soil transmitted helminths (STH) infestations constitute a considerable disease burden in schoolchildren [4–6]. These parasitic infestations are reported to have similar geographic distribution at various epidemiological settings in Africa [7,8]. In these settings, asymptomatic malaria parasitaemia has been reported with a higher rate in schoolchildren, compared to the general population including pregnant women, making them reservoir of infection [9–13]. Furthermore, malaria in schoolchildren accounts for about 13–50% of all school absenteeism, causes anaemia that also impairs the cognitive development [7]. In addition, coinfections with malaria and STH are reported mostly in the tropics and the populations at high risk are school age children [5] and pregnant women [8].

Studies have demonstrated that asymptomatic infections are likely to harbour gametocytes at microscopic and sub-microscopic levels thereby contributing to persistent malaria transmission [14]. The prolonged carriage of *P*. *falciparum* triggers the development of acquired immunity that controls blood-stage parasitaemia, thereby reducing clinical symptoms and life-threatening complications in older children and adults [15]. However, if untreated, asymptomatic *Plasmodium* parasitaemia persists, and maintains malaria-induced inflammation that is commonly associated with iron deficiency anaemia (IDA) due to impaired intestinal iron absorption, impaired iron release from hepatocytes, and impairment of the recycling of iron derived from phagocytosis of parasitized red blood cells [7,16]. Approximately 85 million school-aged children of sub Saharan Africa are affected by malaria related anaemia [7]. Co-morbidities with STH complicates the problem even further as they as well contribute to anaemia, nutritional deficiencies and cognitive impairment [17,18]. Less is known on the attributable fraction of asymptomatic malaria infection on anaemia, and possibly predict theimpact of malaria intervention on anaemia, cognitive and psychomotor functions in schoolchildren.

STH control has been widely introduced in schools where, annual deworming with antihelminths have been successfully implemented. In this, school children receive deworming drugs at schools through their teachers [19–21]. Similar control interventions by the use of drugs for malaria in school children have recently been highly advocated though at different schedules [22,23]. However, use of antimalarial drugs in such endeavour, has to be considered with full understanding of risk of developing antimalarial drug resistance in the targeted population. For example, in north-eastern Tanzania, there are well documented reports of high levels of super-resistant parasites to the antimalarial drug sulfadoxine-pyrimethamine (SP) owing to widespread levels of *P*. *falciparum* dihydropteroate synthetase (*Pfdhps*) and dihydrofolate reductase (*Pfdhfr*) leading to quadruple, quintuple and sextuple mutant parasites [24,25]. Mutations in the *Pfdhps* A581G (>10%) and K540E (90%) were shown to compromise the effectiveness of SP when used as intermittent preventive treatment during pregnancy (IPTp-SP), particularly in Eastern African [26]. There is also lack of recent evidence on the *P*. *falciparum* gene encoding kelch-13 (*Pfkelch-13)* associated with prolonged parasite clearance time and other markers of partners drugs in the artemisinin-based combination therapy (ACTs) [27]. Understanding the epidemiology of validated antimalarial resistant markers, would help in shaping any malaria intervention strategy such as IPT for school children as suggested elsewhere [23].

We thus, conducted a study to determine the risk factors for asymptomatic malaria and their association to anaemia. We explored (1) the association of asymptomatic malaria infection and anaemia on cognitive and psychomotor functions and (2) the prevalence of antimalarial drug resistance in school-aged children living in high endemic areas. We then discuss possible interventions to overcome malaria and anaemia in schoolchildren.

## Materials and methods

### Study area

**Fig 1:**
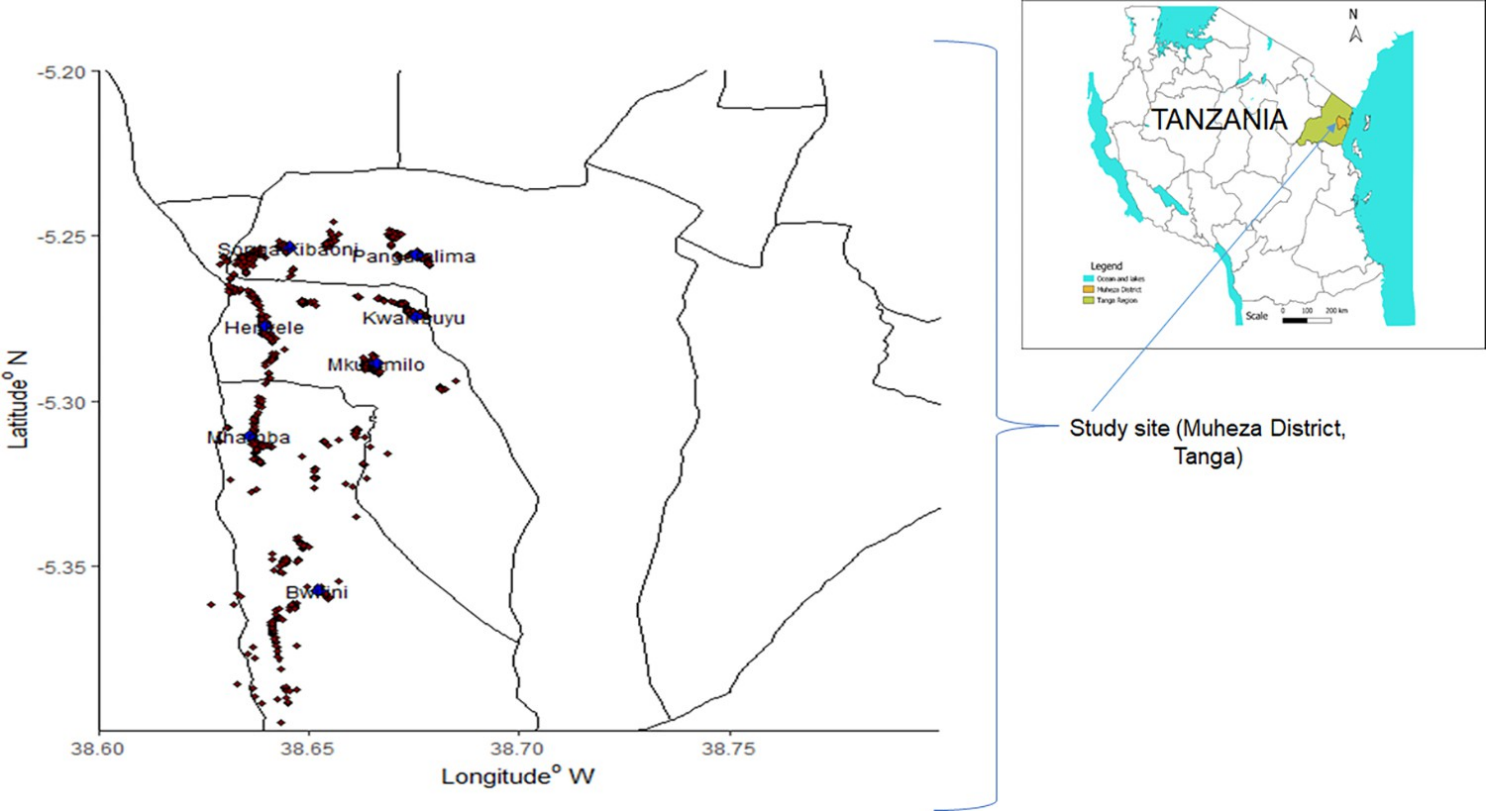
Muheza District map showing study area (schools) and respective households with pupils’ location. Schools are pointed as blue dots and household enumerated in red dots.

The survey was conducted in Muheza District, Tanga Region, north-eastern Tanzania (Fig 1), where, malaria transmission occurs throughout the year with two seasonal peaks following the long rainy season from July to August and the short rainy season from December to January [28]. Malariometric surveys conducted in 2014 at Muheza showed mean malaria prevalence was higher among children aged 5–14 years old, compared to <5 years old (38% vs. 18% and 39% vs. 34% after the short and long rains, respectively) [28]. In this survey, the participants were recruited from seven selected primary schools namely; Pangamlima, Songa Kibaoni, Heinkele, Kwakibuyu, Mhamba, Bwitini and Mkulumilo, located in villages with high malaria prevalence in Muheza District following a survey conducted in 2014 [28]. In the study area, as well as national wise, there has been programmatic provision of drugs against STH and schistosomiasis each year under the national neglected tropical disease control programme (NTDP). However, there has never been any school-based malaria control effort apart from community bednet distribution that may sometimes include distribution in schools (personal conversation with NMCP).

### Study design

This study was a comprehensive baseline survey, within the scope of a randomised, controlled trial enrolling asymptomatic schoolchildren and investigating the effectiveness and safety of two antimalarial drugs namely Dihydroartemisinin-piperaquine (DP) and Artesunate-amodiaquine (ASAQ) in preventing malaria related morbidity in schoolchildren living in high endemic areas. The trial design and method of the intervention have previously been detailed in the study protocol [29] (also registered on clinical trials.gov -NCT03640403). A comprehensive survey was conducted from Feb 25^th^ to April 29^th^ 2019, covering eligible schoolchildren and their respective households. Asymptomatic schoolchildren were recruited following consent from their parents and an assent for children aged 11 years and above. Children of class 5 or below aged 5–15 years old were recruited for clinical evaluation and later enrolled in the clinical trial. Children of class 6 or 7 were excluded because they would graduate school before the trial ends. Consented parents were visited at their households where socioeconomic and demographic information including bednet ownership and use were obtained using a structured questionnaire designed on REDCap software [30]. Geo-reference data for key parameters were mapped at the household level by means of a geographic information system (GIS). At schools, enrolled children were clinically examined for fever and malnutrition. Reporting of the current study has been verified in accordance with the Strengthening the Reporting of Observational Studies in Epidemiology (STROBE) checklist.

### Recruitment

A school census was conducted in all seven primary schools selected for the study. Prior to recruitment, the study team held school committee meetings [for a brief study information to the school and village leaders and representatives before going to the general public], and then a school meeting involving parents /guardians and village leaders was held in a respective school. The study was explained to all parents / guardians who attended the meeting. Parents or guardians with children aged 5–15 years old and who were in class 5 or below, were invited to join the study. These were further informed of the study, and allowed to ask questions whenever they wanted. A written informed consent was signed after interested parents /guardians agreed their children to participate in the study. In addition, an assent was also obtained from children aged 11 years and above.

### Clinical and lab assessment for malaria, STH, schistosomiasis and antimalarial drug resistance

Clinical assessment was conducted at schools involving schoolchildren whose parents / guardians gave consent. Height and weight were measured and axillary temperature was digitally recorded. Thick and thin blood smears (to measure malaria parasitaemia using microscope-Olympus CX23) were obtained from all participants prior to treatment. Haemoglobin concentration was measured using a haemoglobinometer (HemoCue AB, Sweden). Febrile and or anaemic participants were treated by the study team onsite and those needing hospitalisation were referred to a district hospital.

Stool and urine samples were also collected. A stool sample was used to determine the prevalence (defined as adults or eggs) of STH (covering hookworms, *Ascaris lumbricoid*e*s* and *Trichuris trichiura*) and *Schistosoma mansoni* infestation in school-aged children determined by duplicate Kato-Katz thick smears technique as reported elsewhere [31,32]. Urine was first assessed for blood in urine using dipstick test, and then centrifuged. The urine sediments were processed for Kato-Katz thick smears to determine eggs for *S*. *haematobium*. Technical aspects of blood testing and other assessment are detailed on the study protocol [29].

Finger-prick blood (dried blood spot-DBS) was prepared on Whatman 3 M filter paper for the detection of markers of drug resistance, where, parasite DNA was extracted from DBS by the QIAamp 96 DNA blood kit (including the Qiagen supplemental protocol for DBS) according to manufacturer’s instructions (Qiagen, Aarhus, Denmark), and stored at -20°C until further analyses. All extracted samples were analysed by *Plasmodium-*specific PCR for the detection of parasite DNA [33]. All *Plasmodium-*positive samples were further analysed for the presence of *P*. *falciparum* molecular resistance markers, mainly *Pfdhfr*, *Pfdhps*, *the P*. *falciparum* multi-drug resistance gene 1 *(Pfmdr-1)*, the chloroquine resistance transporter (*pfcrt*) and the *P*. *falciparum* exonuclease (*Pfexo*). This was done by the Illumina targeted amplicon sequencing technique as previously described [34].

### Psychomotor and cognitive function

Psychomotor and Cognitive function were assessed using a ‘20 metre Shuttle run’ and a test of everyday attention for children (TEA-Ch), respectively. Psychomotor function was determined by VO2max, determined as a measure of how much oxygen the body uses during exercise at a maximum effort [35]. A 20 meter shuttle run method was deployed [35,36]. The test was conducted at school on a clear and level playing field during school hours as was done elsewhere [37], to maximize convenience and minimize disruption to the school day programme. Nine to 12 children were tested at any one time. For every three children, one observer was ascribed to ensure adequate monitoring of their performance. Careful instructions were given and the study team performed a brief demonstration of the test before testing. All children were kept well hydrated, and water and sugary snacks were made available as was done elsewhere [37]. The study participants for ease of identification wore coloured, light scarfs. As described in the study protocol [29] during this test, children ran continuously between two lines apart turning when signalled to do so by recorded beeps and a “shuttle” was defined as a run between one line to another. Each fitness score was then translated into VO2max (mL kg−1 min−1) by calculation using a formula by Leger *et al*,1988 [38].

Cognitive function was determined by a measure of sustained attention method adopted from Clarke *et al* [39]. We selected enrolled pupils from class 4 and 5, who we presume were old enough to understand the cognitive test instruction. We evaluated sustained attention using two code transmission tasks, adapted from the TEA-Ch battery by Manly T. *et al* [40]. Tests involved listening to a pre-recorded list of digits read aloud at the speed of one per second. Children were required to listen out a ‘code’. One could score 0 to a maximum of 40 scores.

### Statistical analysis

The sample size was estimated on the basis of the intervention described in the main study [29], where a sample of 1602 schoolchildren was expected. During the surveys, data were entered directly into the REDCap platforms using mobile REDCap tools [29,30,41]. Paper based data (from laboratory results) were double entered and verified in Microsoft Access 2010 (Redmond, WA, USA), while consistence checks and analyses were done using STATA software version 12.0 (StataCorp LP, TX, USA). Principal component analysis (PCA) was used to categorize children and their respective households into different socioeconomic statuses (SES). Household socioeconomic scores were grouped into two quantiles defined as low, and high. Variables which were considered in the PCA were: type and building material of the house (roof, walls, floor, ceiling), toilet type, presence of electricity, ownership of radio, mobile phone, bicycle, motorbike, vehicle, ratio of number of sleeping bedrooms, occupation of head of household as well as number of animal and size of land owned by the family [42]. Malaria parasite prevalence was calculated as the number of children with any parasites (irrespective of species) on thick blood smear divided by the total number of children enrolled [43]. Anaemia was defined using the World Health Organization (WHO) age-specific cut off points for haemoglobin (< 11.5 g/dL for children 6 to < 12 years of age,< 12.0 g/dL for those 12–14 years of age, <13 g/dL and <12 g/dL for male and female children aged 15 years, respectively) [43,44]. Body mass index (BMI) was calculated as (weight(Kg)/height(m)^2^), the respective anthropometric index z-score including that of height for age (HAZ) and weight for age (WAZ) was calculated using the ‘*egen’* STATA function as described elsewhere [43,45]. Children were classified as underweight / wasted if they were less than two standard deviations (SD) below the reference mean [43]. The outcomes of interest were *Plasmodium* parasitaemia regardless of species, anaemia, STH and schistosomiasis infestation. Cognitive and psychomotor function scores were also outcomes of interest given their relationship with anaemia or malaria in such age group [20,29,43,46,47].

For the general characteristic table, ninety-five percent binomial confidence intervals (CI) were estimated for the difference in proportions or means between those who were clinically evaluated and those who were excluded from the successive analysis, using T-test. Standard deviations were presented for means. The same was done for the general clinical characteristics but stratified by sex. Univariate associations between *Plasmodium* infection or anaemia and potential risk factors were assessed using logistic regression and all variables showing a significant association were included into a multivariate logistic regression model as noted elsewhere [43]. Logical model building using both forward and backward elimination approach was used to generate minimum adequate model using a 5% significance level. The model was considered a best fit, if it had significant maximum likelihood estimation, high level of estimate classification in terms of sensitivity and specificity (percent correctly classified), and also had high area under the curve in a ROC curve produced following logistic regression model. Goodness of fit was tested using Hosmer-Lemeshow chi^2^. Attribution fraction (AF) of variables with significant association with either malaria or anaemia was determined using respective adjusted odds ratio (aOR) in the model and was then used to determine respective population attributable fraction (PAF) using methods described elsewhere [48–52]. Where, AF = [aOR-1 / aOR)] and PAF = [AF x Proportion of a factor in a population].

To investigate the association between asymptomatic *Plasmodium* infection and cognitive or psychomotor function, two sets of analyses, one for psychomotor function expressed in “VO2max” scores and the second for cognitive function expressed as sustained attention (score 0–40) were undertaken. The effect of explanatory variables was quantified by mean differences in test performance scores using univariate and multivariate linear regression [53]. Since only a subgroup of pupils participated in the cognitive and psychomotor function tests, bootstrap methods were used to account for non-normality of the scores. Since schools could represent a cluster effect, all models included schools to adjust for its effect on the measured outcome. On this, a school whose children have the lowest mean location altitude was considered a reference group in each model.

### Ethical approval and consent to participate

The study obtained ethical clearance from the Medical Research Coordinating Committee (MRCC, Tanzania) with approval number NIMR/HQ/R.8a/Vol.IX/2818 and NIMR/HQ/R.8c/Vol.I/668 (for amendment) also NIMR/HQ/R.8c/Vol.I/1276 for ethical clearance extension. We obtained regulatory approval from the Tanzania Medicines and Medical Devices Authority (TMDA) with approval number TFDA0017/CTR/0018/07. Each participant signed an informed consent/assent to join the study. The study also obtained permissions from different levels of local government including local school committees.

## Results

**Fig 2:**
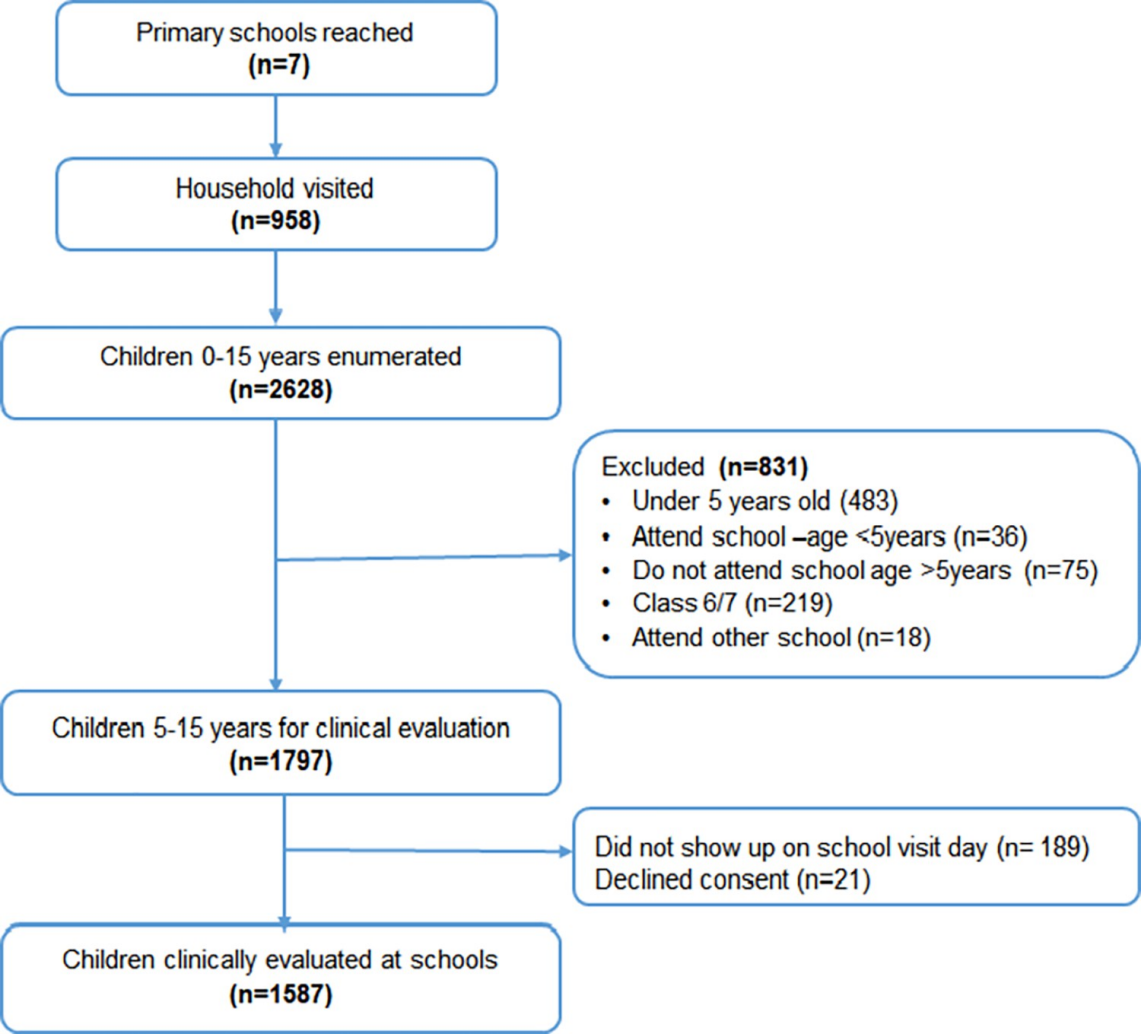
Study flow chart showing recruitment process for epidemiological assessment on malaria and anaemia in schoolchildren participating in the study in Muheza, north-eastern Tanzania.

A flow diagram (Fig 2) summarizes the study approach. The study team reached 958 households from the seven primary schools selected for the study. These households hosted children whose parents or guardians consented for the study. In the households, there were about 2,628 children aged 15 years and below, enumerated for socio-demographic and economic assessments. The 2,628 children were descriptively analysed as shown on Table 2. For the clinical assessment conducted at schools, 831 children did not fulfil the inclusion criteria due to various reasons listed on the Fig 2. The 831 children were only excluded from the outcome analysis based on clinical assessment. About 1,797 children set for clinical screening; however, 189 of them did not show up on the clinical assessment visit, while 21 declined consent, resulting in 1,587 children available for testing and further recruitment onto the clinical trial.

### Characteristics of schoolchildren and their households

A total of 2,628 children were enumerated in households that had consented for the study, of these 2,109 (80%) were children of school age (i.e. 5 to 15 years old). The mean age overall was 8 years (SD = 4), (Table 1). Male children were 52% of all children though the difference with female children (48%) was not statistically significant. Overall, 38% of children reported history of malaria episode in the last month. Among children who reported malaria last month, 76% had confirmed malaria by microscopy or malaria rapid diagnostic test (mRDT). The common malaria treatment place was at a health facility (70%) followed by a drug shop (29%). Of all children, 81% had slept under a bednet in a previous night and about 90% of the bednets were long lasting insecticide treated nets (LLIN). However, over 80% of the bednets owned were torn with holes, also verified by the study team during the interview. About 45% of children reported to have received anthelminthic medication in the past 6 months, of whom the majority were those who attend school (OR = 8.02, 95%CI 6.09–10.56, P<0.0001). Most children had parents or guardians with a primary school education (82%), lived in houses with open eaves (81%), and had livestock living inside the house (75%). The mean altitude for children’s household was 226 (SD = 34.9) meters above sea level. The mean number of people in a household was 6 (SD = 2.2), mean number of children per household was 3 (SD = 1.5) and the mean number of sleeping space per household was 2 (SD = 1.0) (Table 1). All these characteristics had no significant difference between males (boys) and females (girls) children.

**Table 1:** Description of children and their households in Muheza, north-eastern Tanzania.

Variable	Category	All (N = 2,628)	Involvement in clinical evaluation		p-value for differences
		mean or %	Yes (N = 1,587)	No (N = 1,041)	
Mean age (SD)	all, mean (SD)	8.0 (4.0)	9.1 (2.6)	6.4 (5.0)	**<0.01**
Sex	Male, n (%)	1377 (52.4)	837 (52.7)	540 (51.9)	0.66
	Female, n (%)	1251 (47.6)	750 (47.3)	501 (48.1)	
Had malaria last month	n (%)	1003 (38.2)	669 (42.2)	334 (32.1)	**<0.01**
Malaria tested last month (confirmed), n (%)		759 (76.1)	504 (75.9)	255 (76.4)	0.88
Common malaria treatment place	Health facility, n (%)	638 (70.3)	375 (70.0)	263 (70.7)	0.81
	Drug shop, n (%)	260 (28.6)	154 (28.7)	106 (28.5)	0.94
	Traditional, n (%)	10 (1.1)	7 (1.3)	3 (0.8)	0.47
Slept under a bednet last night, Yes %		2135 (81.2)	1252 (78.9)	883 (84.8)	**<0.01**
Net is LLIN, n (%)		1907 (89.3)	1130 (90.3)	777 (88.0)	0.10
Net with holes, n (%)		1738 (81.4)	1037 (82.8)	701 (79.4)	**0.04**
Received anthelminthic medication last 6 months, n (%)		1183 (45.0)	876 (55.2)	306 (29.4)	**<0.01**
Household location altitude, Mean(SD) -meters		225.6 (34.9)	223.7 (34.7)	228.4 (34.9)	**0.01**
Household Socioeconomic status, Low, n (%)		1314 (50)	814 (51.3)	500 (48.0)	0.10
	Secondary or high, n (%)	167 (6.6)	93 (6.1)	74 (7.3)	0.24
Parent’s level of education	Primary, n (%)	2072 (81.7)	1239 (81.5)	833 (82.0)	0.74
	None, n (%)	298 (11.8)	189 (12.4)	109 (10.7)	0.19
Houses with eaves open, n (%)		2013 (81.4)	1210 (81.3)	803 (81.4)	0.94
Livestock live inside house, n (%)		1385 (75.6)	829 (75.2)	556 (76.2)	0.62
Number of people in a HH, Mean (SD)		6.1 (2.2)	5.9 (2.1)	6.4 (2.3)	**<0.01**
Number of children in a HH, Mean (SD)		3.5 (1.5)	3.3 (1.5)	3.7 (1.5)	**<0.01**
Number of rooms in HH, Mean (SD)		3.4 (1.3)	3.4 (1.3)	3.4 (1.3)	0.59
Number of rooms for sleeping, Mean (SD)		2.3 (1.0)	2.3 (2.2)	2.4 (2.3)	0.11

*HH = House hold*, *LLIN = Long lasting Insecticide treated net*, *STH = Soil Transmitted Helminths*, *SD = Standard Deviation*.

Deriving from Fig 1, Table 1, further shows general characteristics of children stratified by whether they were involved in the subsequent sub analysis [i.e. those clinically evaluated (n = 1,587)] or those who were not [i.e. those excluded (n = 831) plus those who missed clinical evaluation (n = 189+21)]. The significant differences (p<0.05) in baseline characteristics between the two groups (i.e. clinically evaluated vs not clinically evaluated, respectively) were notably on: mean age (9 vs 6 years), history of malaria infection last month (42% vs 32%), slept under a bednet in the last night (79% vs 85%), having a torn bednet with holes (83% vs 79%), received STH medication in the last 6 months (55% vs 29%), mean household location altitude (223.7 vs 228.4 meters), mean number of people in a household (5.9 vs 6.4) and mean number of children in a household (3.3 vs 3.7).

The supplementary tables (S1 and S2 Tables) provide further information on children who did not show up for clinical assessment (n = 189) together with those who did not consent for clinical evaluation (n = 21) (Fig 2). These essentially were not different to participants who were clinically assessed. Also on comparing children by age groups (5–15 years vs under-fives) the respective significant differences were; history of malaria last month (40% vs 31%), slept under a bednet in the last night (79% vs 89%), having a torn bednet with holes (83% vs 74%) and received anthelminthic medication last 6 months (53% vs 12%) (See S1 and S2 Tables).

### Clinical, parasitological, cognitive and psychomotor assessments

At schools, children of class 5 or below, aged 5 to 15 years old were recruited for the study, of these 55% were aged 5 to 9 years old (Table 2). In total, 28.0% were wasted or underweight. Boys had a higher prevalence of stunting compared to girls (diff 5.2% 95%CI 1.14–9.16, *p = 0*.*01*). Overall, prevalence of anaemia (Hb<12g/dL) was 49.8% (95%CI 24.2–28.7), out of which 60% was moderate anaemia (Hb between 8.0–10.9g/dL), and 37% were with mild anaemia (Hb between 11.0–11.9g/dL for children aged 12 years and above, or 11.0–11.4g/dL for children below 12 years old, and 11.0–12.9g/dL for boys aged 15 years. 3.2% had severe anaemia (Hb<8.0g/dL). Asymptomatic malaria prevalence was 26% (95%CI 24.2–28.7), at an arithmetic mean parasitic density of 4,264.7 asexual stages /μL (95% CI 1,352.4–7,177.0) and the geometric mean of 752.7 asexual stages /μL (95% CI 637.8–888.3), for *P*. *falciparum*. Other species (*P*. *malariae and P*. *ovale*) and gametocytes had lower parasitaemia levels as shown in Table 2. Boys had a higher malaria prevalence than girls, proportion difference was 6.4% 95%CI 1.91–10.93, *p = 0*.*005*. Only 2.3% (n = 34) of children were found to be febrile (temperature > = 37.5°C) or had a history of fever in the past 48 hours of the study visit, of whom half (53%) tested positive for malaria. STH infestation was only in 1% of the population, and the most common infestation was hookworm (99%), at an average egg count of 90.3 (SD = 73.7). Blood in urine was found in 15% of schoolchildren (N = 226). Urine sediments revealed *S*. *haematobium* in 8.5% of population at a mean egg count of 19.1 (SD = 21.3). Boys were more affected than girls (diff in prevalence 3.8%, 95% CI 1.02–6.57, *p = 0*.*0073*). Children of class 4 and 5 performed the cognitive and psychomotor function tests. The mean score on sustained attention test (TEACh) was 15.7 (SD = 15.1) with no significant difference between boys and girls. On psychomotor function test, boys dominated at a mean difference of 1.74 (95%CI 1.35–2.13) and 3.67 (95%CI 2.62–4.73) for shuttle level and VO2max, respectively (P<0.001).

**Table 2:** General clinical characteristics of schoolchildren in Muheza, north-eastern Tanzania.

Variable	Category	Overall (N = 1587) n (mean or %)	Sex	
			Male (N = 837)	Female (N = 750)
Age group distribution	10 to 15 years, n (%)	714 (45)	397 (55.6)	317(44.4)
	5 to 9 years, n (%)	873 (55)	440 (50.4)	433(49.6)
Mean age per age group (SD)	10 to 15 years	11.5 (1.4)	11.6 (1.4)	11.5 (1.3)
	5 to 9 years	7.2 (1.4)	7.2 (1.3)	7.1 (1.4)
Household Altitude, mean in meters (SD)		223.7 (34.7)	223.0 (33.2)	224.4 (36.4)
Mean weight in kg (SD)		24.5 (6.9)	24.6 (6.6)	24.5 (7.3)
Mean height in cm (SD)		126.9 (10.7)	127.0 (10.2)	126.8 (11.2)
Anthropometric indicators of malnutrition				
	WAZ <-2zscore, n (%)	441 (27.8)	243 (29)	198 (26.4)
	HAZ <-2 zscore, n (%)	333 (21.0)	196 (23.4)	137 (18.3)
	BMI <-2zscore, n (%)	448 (28.2)	231 (27.6)	217 (28.9)
Fever history or Temperature > = 37.5°C, n (%)		36 (2.3)	19 (2.3)	17 (2.3)
Fever or T> = 37.5°C with positive malaria, n (%)		18 (52.9)	11 (61.1)	7 (43.7)
Anaemia (WHO criteria per age)	All n (%)	791 (49.8)	434 (51.8)	357 (47.6)
	Mild n (%)	290 (36.7)	157 (36.2)	133 (37.3)
	Moderate n (%)	476 (60.2)	263 (60.6)	213 (59.7)
	Severe n (%)	25 (3.2)	14 (3.2)	11 (3.1)
Parasitic infection				
Asymptomatic malaria prevalence, n (%)		388 (26.4)	229 (29.4)	159 (23.0)
Asymptomatic (Pf) density, n (geometric mean)		381 (752.7)	225 (872.6)	156 (608.1)
Asymptomatic (Pf) density, n (mean)		381 (4264.7)	225 (3841.5)	156 (4875.1)
Asymptomatic (Pm) density, n (mean)		32 (324.5)	25 (366.1)	7 (176.0)
Asymptomatic (Fg) count, n (mean)		20 (118.4)	15 (129.2)	5 (86.0)
Asymptomatic (Po) density, n (mean)		2 (112)	1 (128)	1 (96)
STH prevalence (%)		17 (1.1)	12 (1.5)	5 (0.7)
STH-mean egg count n (mean)		17 (90.3)	12 (97.9)	5 (72.0)
RBC and WBC in urine n (%)		226 (14.6)	125 (15.2)	101 (13.8)
*S*. *haematobium* prevalence n (%)		131 (8.5)	84 (10.2)	47 (6.5)
*S*. *haematobium* egg count, n (mean)		131 (19.1)	84 (23.0)	47 (12.2)
Cognitive and psychomotor functions (class 4 and 5 only)				
	Shuttle level, n (mean)	316 (6.1)	168 (7.0)	148 (5.2)
	VO2max, n (mean)	316 (48.5)	168 (50.3)	148 (46.6)
Cognitive score, n (mean)		313 (15.8)	166 (16.9)	147 (14.6)

*WAZ = weight for age z-score*, *HAZ = Height for age z-score*, *BMI = Body Mass Index*, *Pf = Plasmodium falciparum*, *Pm = Plasmodium malariae*, *Po = Plasmodium ovale*, *Fg = Plasmodium falciparum gametocyte*, *STH = Soil Transmitted Helminths*, *SD = Standard Deviation*, *RBC = Red Blood Cells*, *WBC = White Blood cells*.

There was variation across schools for different characteristics (Table 3). Heinkele was a school whose participants were located at a higher altitude (mean of 258.4 meters above sea level) and had the lowest malaria infection prevalence at 19%. Generally, across schools, participants who had asymptomatic malaria infection resided at a location with slightly lower mean altitude of 218.4 meters [mean difference of 7.2 meters, 95% CI 3.0–11.4, *p = 0*.*0008*]. However, participants at Pangamlima located at a mean altitude of 213.3 meters were not the least in altitude location but had the highest malaria prevalence over all schools (40%). STH was less prevalent in the study area, only 1% of the population had hookworm infestation. Overall, Kwakibuyu had high anaemia, STH and schistosomiasis infestations, 75%, 2.9%, and 30.3%, respectively. Songa Kibaoni primary school had the least anaemia prevalence at 24% and had a higher mean cognitive score (27.3) of all schools. Bwitini primary school had the least mean cognitive score of 11.8. The median age of all children in each school was similar at 9 years old. The VO2max was essentially stable across schools. Other variations are as seen on the Table 3.

**Table 3:** General clinical characteristics of children per respective schools in Muheza, north-eastern, Tanzania.

School name	Observations N	Mean Altitude,(range)		Median age years (IQR)	Low SES, N (%)	Malaria, N (%)	Anaemia, N (%)	STH, N (%)	Schistosomiasis, N (%)	Stunting N, (%)	Cognitive score, N (mean)	VO2max score, N (mean)
Pangamlima	179	213.3	(176.9–251.9)	9 (7–12)	179 (53.6)	160 (40.0)	179 (35.2)	177 (0.56)	172 (0.58)	179 (22.4)	50 (14.7)	51 (48.0)
Songa Kibaoni	265	241.5	(179.8–290.9)	9 (7–11)	265 (49.1)	237 (22.4)	265 (24.5)	261 (0.77)	263 (23.57)	265 (22.3)	45 (27.3)	46 (46.8)
Heinkele	283	258.4	(195.2–338.7)	9 (8–11)	283 (53.4)	269 (19.0)	283 (47.7)	279 (1.43)	279 (2.15)	283 (21.6)	33 (13.3)	34 (50.2)
Kwakibuyu	176	189.6	(161.1–243.2)	9 (7–11)	176 (51.7)	167 (35.3)	176 (75.6)	175 (2.86)	175 (30.29)	176 (29.0)	42 (17.1)	42 (46.5)
Mhamba	241	244.3	(134.1–296.6)	9 (7–11)	241 (53.5)	228 (20.2)	241 (64.3)	238 (0.42)	235 (0.43)	241 (11.6)	37 (17.7)	37 (49.4)
Bwitini	241	195.8	(91.2–272.3)	9 (7–11)	241 (45.2)	220 (23.6)	241 (49.0)	231 (0.87)	229 (0.00)	241 (20.3)	56 (11.8)	56 (49.2)
Mkulumilo	202	203.1	(158.9–257.9)	9 (7–11)	202 (53.5)	190 (33.2)	202 (60.4)	196 (1.02)	196 (4.08)	202 (22.3)	50 (10.4)	50 (49.6)
All schools	1,587	223.7	(91.2–338.7)	9 (7–11)	1,587 (51.3)	1,471 (26.4)	1,587 (49.8)	1,557 (1.09)	1,549 (8.46)	1,587 (21.0)	313 (15.8)	316 (48.5)

*SES = socioeconomic status*, Stunting* = <-2 Height for age z-score, *IQR = Inter quantile range*, *STH = Soil Transmitted Helminths*.

### Risk factors and their attribution to malaria infection in schoolchildren

Age group (10–15 years), low socioeconomic status, low level of parents or guardian education (primary or none), not sleeping under a bednet, fewer rooms and increased number of children (overcrowding) in a household were significantly associated with high odds of malaria infection (Table 4). Children with anaemia had an adjusted 2.6 odds (95% CI 1.9–3.4, P<0.01) of having malaria compared to those without anaemia, and malaria attributed to 61% (AF) of anaemia in children with malaria. Children who came from households with low and moderate socioeconomic status attributed to 11% and 12%, respectively to malaria infection to the study population. Boys had 1.4 odds (95% CI 1.0–1.7, *p = 0*.*01*) of malaria infection compared to girls, but became non-significant on multivariate analysis. Children aged 10–15 years old had 1.4 adjusted odds (95%CI 1.0–1.8, *p = 0*.*03*) of having malaria parasite compared to those aged 5–9 years, this group attributed to 28% of malaria infection per age group, and attributed to 12% of malaria infection in the population. Not sleeping under a bednet attributed to 34% of having malaria infection (Table 4). Having parents or guardian with primary school or lower level of education attributed to 55% of having malaria infection which was corresponding to 45% of malaria in the population. Having more number of children in a household was associated with an increase in malaria infection, it attributed to 13% of infection in the population. Children from household with less number of rooms in a household were associated with more malaria infection, this attributed to 11% of malaria in a population. Children from Mkulumilo, Kwakibuyu and Pangamlima primary schools were also significantly associated with increased odds of malaria infection; their average attribution to the population (PAF) was 7%, 6% and 8%, respectively. STH, *S*. *haematobium* infestation, open eaves and nutritional status were not associated with malaria infection.

**Table 4:** Univariate and multivariate analysis of factors associated with asymptomatic malaria infection among primary schoolchildren in Muheza, north-eastern Tanzania.

		n/N with *Plasmodium* infection	Univariate analysis			Multivariate analysis				
Variables			Crude odds ratio (95% CI)	P-value	Adjusted odds ratio (95% CI)	P-value	AF	PAF
Sex	Female	159/692								
	Male	229/779	1.40	(1.10–1.77)	0.01	1.27	(0.97–1.66)	0.08		
Age group	5–9 years	182/808								
	10–15 years	206/663	1.55	(1.23–1.96)	<0.001	1.38	(1.04–1.84)	0.03	0.28	0.12
Nutritional status										
	WAZ <-2zscore	121/416	1.21	(0.94–1.56)	0.14					
	HAZ <-2 zscore	110/303	1.82	(1.39–2.39)	<0.001	1.15	(0.82–1.62)	0.41		
	BMI <-2zscore	102/427	0.83	(0.64–1.08)	0.17					
Anaemia		256/732	2.47	(1.94–3.14)	<0.001	2.56	(1.91–3.42)	<0.001		
STH infestation		5/16	1.27	(0.44–3.68)	0.66					
*S*. *haematobium* infestation		41/122	1.46	(0.98–2.17)	0.06	1.39	(0.85–2.25)	0.19		
Number of children in a HH		363/1354	1.15	(1.06–1.25)	<0.001	1.17	(1.07–1.28)	<0.001	0.15	0.13
Few rooms in a HH		371/1396	1.13	(1.03–1.24)	0.01	1.12	(1.01–1.25)	0.04	0.11	0.10
Household Altitude		363/1007	0.99	(0.99–1.00)	0.001	1.00	(1.00–1.00)	0.57		
SES	Higher SES	90/464								
	Moderate SES	136/479	1.65	(1.22–2.23)	<0.001	1.60	(1.13–2.26)	0.01	0.38	0.12
	Low SES	162/528	1.84	(1.37–2.47)	<0.001	1.43	(1.12–2.07)	0.047	0.30	0.11
Head of household education level										
Secondary or above		12/86								
	Primary	307/1141	2.27	(1.22–4.24)	0.01	2.22	(1.09–4.52)	0.03	0.55	0.45
None		56/178	2.83	(1.42–5.63)	0.001	2.30	(1.04–5.07)	0.04	0.57	0.07
Eaves open		302/1128	1.03	(0.75–1.41)	0.83					
Not sleeping under a bednet		116/318	1.86	(1.42–2.43)	<0.001	1.52	(1.12–2.07)	0.01	0.34	0.07
School*	Heinkele	51/269								
	Mhamba	46/228	1.08	(0.69–1.69)	0.73	0.96	(0.57–1.60)	0.87		
	Songa Kibaoni	53/237	1.23	(0.80–1.90)	0.35	1.55	(0.93–2.59)	0.09		
	Bwitini	52/220	1.32	(0.86–2.05)	0.21	1.45	(0.80–2.65)	0.22		
	Mkulumilo	63/190	2.12	(1.38–3.26)	0.001	2.03	(1.15–3.64)	0.02	0.51	0.07
	Kwakibuyu	59/167	2.34	(1.50–3.63)	0.001	2.03	(1.05–3.95)	0.04	0.51	0.06
	Pangamlima	64/160	2.85	(1.84–4.42)	0.001	3.80	(2.17–6.65)	<0.001	0.74	0.08

*school arranged in ascending order following malaria prevalence, *AF = Attribution fraction = (aOR-1) /aOR*, PAF = Population attribution fraction = [AF*Proportion of a factor in a population]; where aOR is adjusted odds ratio. *SES = Socioeconomic status; Factors used in the adjusted model include anaemia*, *sex*, *age-group*, *socioeconomic status*, *stunting (zhaz)*, *sleep under a bednet*, *education of head of household*, *schistosomiasis infestation*, *location altitude*, *number of children in a household*, *number of rooms in a household*, *and school*.

**Fig 3:**
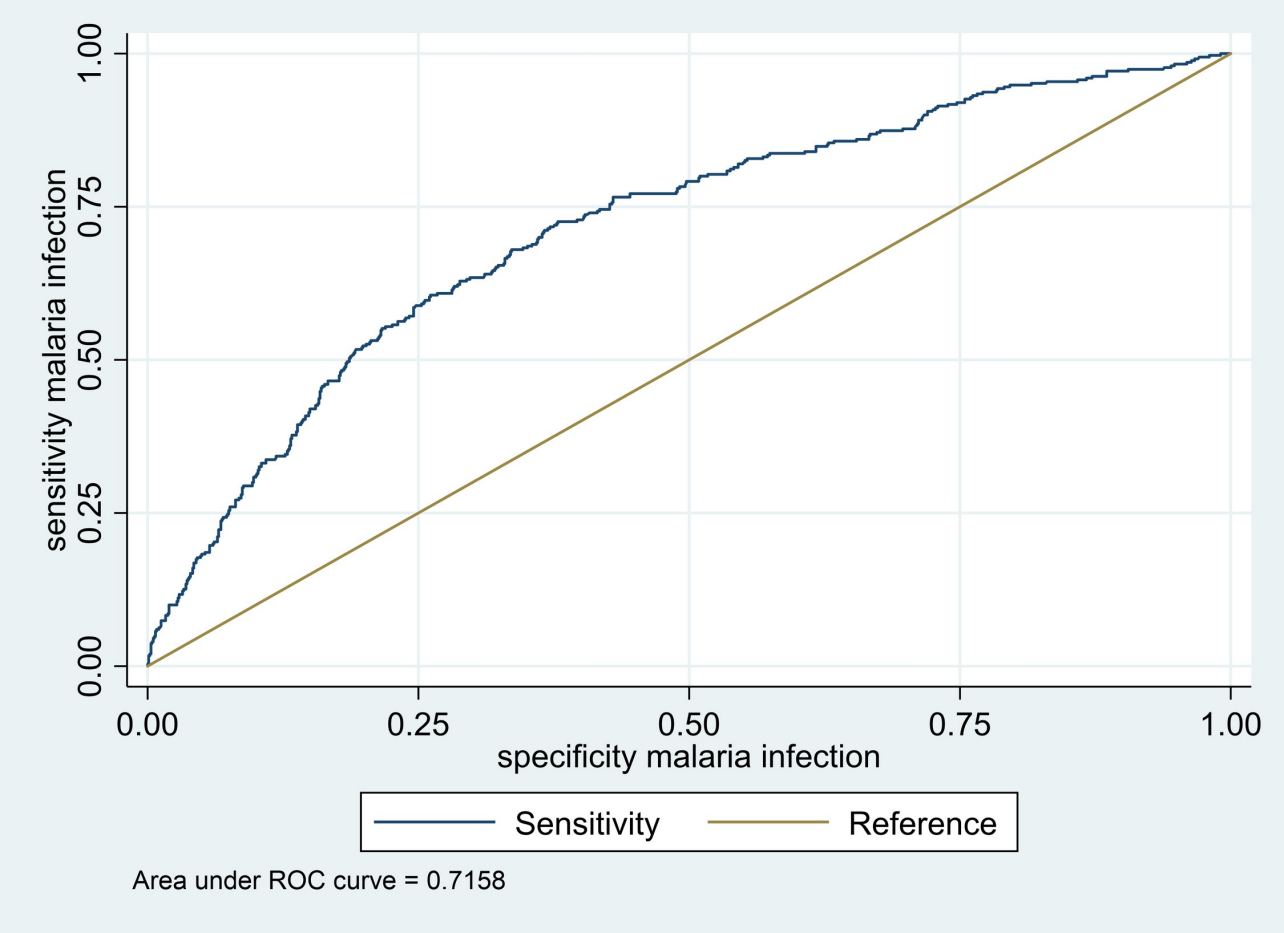
Malaria logistic regression model showing sensitivity and specificity to factors associated with malaria in schoolchildren of Muheza, north-eastern Tanzania. Here presented in a receiver operating characteristics (ROC) curve.

The malaria model was able to predict correctly about various factors in association with malaria infection by 72% depicted as area under the receiver operating characteristic (ROC) curve, developed following the logistic regression (Fig 3).

### Antimalarial drug resistance

**Fig 4:**
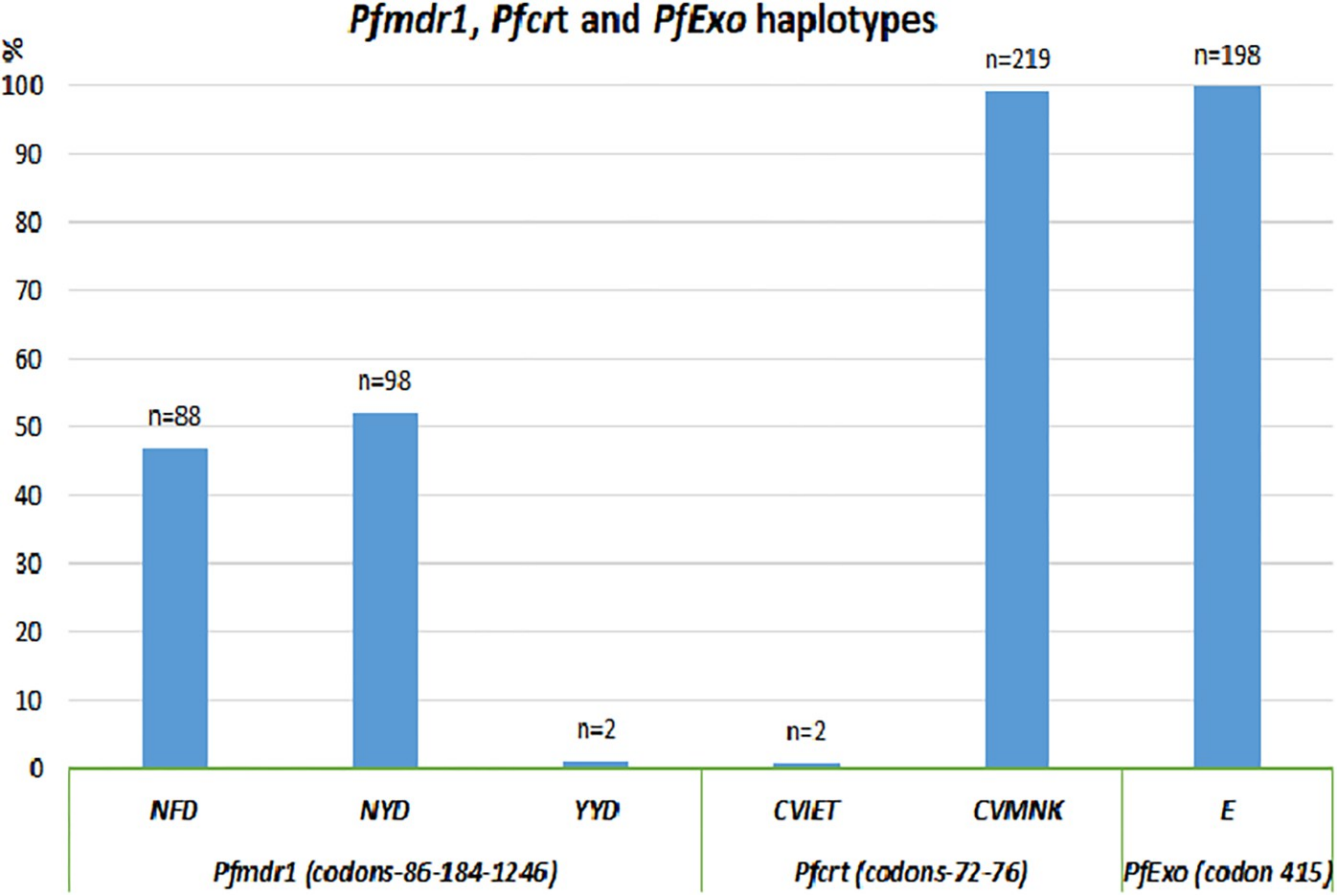
Markers of drug resistance (*Pfmdr1*, *Pfcrt* and *PfExo* haplotypes) from schoolchildren who were parasitised with *P*. *falciparum*, in Muheza, north-eastern Tanzania. *Pfmdr1* = *P*. *falciparum* multi-drug resistance gene 1, *Pfcrt* = *P*. *falciparum* chloroquine resistance transporter, *PfExo* = *P*. *falciparum* exonuclease.

Samples that tested positive for malaria were further analysed for markers of antimalarial drug resistance. The most prevalent *Pfmdr1* 86-184-1034-1042-1246 haplotypes were the NFSND in 47% (n = 88) and the NYSND in 52% (n = 98). The wild type *Pfcrt* haplotypes (codons 72–76, CVMNK) were found in 99.1% (n = 219) of the samples, and CVIET in 0.9% (n = 2). No changes in *Pfexo* was found in codon 415, all samples being wild type E (n = 198) (Fig 4). For 176 infections, it was possible to assemble complete *Pfdhfr/Pfdhps* profiles. Among the 176 infections, 56.3% (n = 99) were carrying the quintuple mutant haplotype, and 33.5% (n = 59) the sextuple mutant haplotype as shown in S1 Table (S3 Table). The results showed a high prevalence of the *Pfdhfr* triple haplotype (n = 244, 91.0%) (51I-59R-108N-164I). The *Pfdhps* double (431I-436S-437G-540E-581A-613A) and triple mutant (431I-436S-437G-540E-581G-613A) were found at prevalence’s of 61.0% (n = 92) and 35.1% (n = 53), respectively (S3 Table). The *Pfkelch-13* mutations were assessed and none of the key validated mutation associated with prolonged parasite clearance was found (see S4 Table).

### Risk factors and their attribution to anaemia in schoolchildren

Children with asymptomatic malaria infection had 2.6 adjusted odds (95% CI 1.9–3.4, P<0.01) of having anaemia compared to those with no malaria infection, and attributed to 61% of anaemia equivalent to 16% attribution of anaemia in the population. This was higher than all other factors in association with anaemia, and was followed by not using bednet and stunting that were attributed to 43% and 40%, respectively. The two factors attributed to 9% and 7% of anaemia in the population, respectively. Children whose parents/ guardian were less educated had high adjusted odds of having anaemia (aOR = 1.75, 95% CI 1.2–2.6, P<0.01) compared with those with education, these attributed to 43% of anaemia in this group and 5% in the study population. *Schistosoma* infestation or low socioeconomic status and location altitude did not have significant association to anaemia in multivariate analysis (Table 5). Children at different schools pointed out on Table 5; appear to have more or less the same population attribution fraction of anaemia to the general population.

**Table 5:** Univariate and multivariate analysis of factors associated with anaemia among primary schoolchildren in Muheza, north-eastern Tanzania.

			Univariate analysis			Multivariate analysis				
Variables		n/N with anaemia	Crude odds ratio (95% CI)		P-value	Adjusted odds ratio (95% CI)		P-value	AF	PAF
Sex	Female	350/733								
	Male	424/818	1.18	(0.96–1.44)	0.11					
Age group	5–9 years	427/854								
	10–15 years	347/697	0.99	(0.81–1.21)	0.93					
Nutritional status										
	WAZ <-2zscore	230/433	1.19	(0.96–1.49)	0.12					
	HAZ <-2 zscore	195/325	1.68	(1.31–2.15)	<0.001	1.51	(1.11–2.04)	0.01	0.34	0.07
	BMI <-2zscore	215/444	0.92	(0.74–1.15)	0.46					
Malaria infection		256/388	2.47	(1.94–3.15)	<0.001	2.54	(1.91–3.37)	<0.001	0.61	0.16
STH infestation		9/17	1.14	(0.44–2.98)	0.78					
*S*. *haematobium* infestation		69/128	1.21	(0.84–1.73)	0.31	1.29	(0.77–2.17)	0.33		
Household Altitude		727/1446	0.99	(0.99–1.00)	<0.001	1.00	(0.99–1.00)	0.87		
Low SES score		416/794	1.23	(1.00–1.50)	0.05	1.08	(0.85–1.38)	0.52		
Head of household education level										
	Primary	584/1209								
Secondary or above		42/92	0.9	(0.59–1.38)	0.62	1.22	(0.74–2.02)	0.43		
None		116/184	1.83	(1.33–2.51)	<0.001	1.75	(1.20–2.55)	0.001	0.43	0.05
Eaves open		603/1187	1.19	(0.91–1.55)	0.20	0.85	(0.62–1.17)	0.33		
Not sleep under a bednet		199/328	1.74	(1.36–2.23)	<0.001	1.68	(1.25–2.25)	<0.001	0.40	0.09
School*										
	Songa Kibaoni	63/260								
	Pangamlima	60/167	1.75	(1.15–2.68)	0.01	1.73	(1.03–2.91)	0.04	0.42	0.05
	Heinkele	134/280	2.87	(1.98–4.15)	<0.001	3.97	(2.53–6.22)	<0.001	0.75	0.14
	Bwitini	114/235	2.95	(2.01–4.32)	<0.001	3.88	(2.31–6.53)	<0.001	0.74	0.11
	Mkulumilo	121/201	4.73	(3.17–7.06)	<0.001	5.39	(3.23–8.99)	<0.001	0.81	0.11
	Mhamba	151/234	5.69	(3.85–8.40)	<0.001	8.33	(5.23–13.27)	<0.001	0.88	0.13
	Kwakibuyu	131/174	9.53	(6.10–14.88)	<0.001	10.23	(5.73–18.27)	<0.001	0.90	0.10

*school arranged in ascending order following malaria prevalence, *AF = Attribution fraction = (aOR-1) /aOR*, PAF = Population attribution fraction = [AF*Proportion of a factor in a population]; where aOR is adjusted odds ratio. *Factors used in the adjusted model includes malaria*, *socioeconomic status*, *stunting (zhaz)*, *not sleeping under a bednet*, *education of head of household*, *schistosomiasis infestation*, *eaves open*, *location altitude and school*.

**Fig 5:**
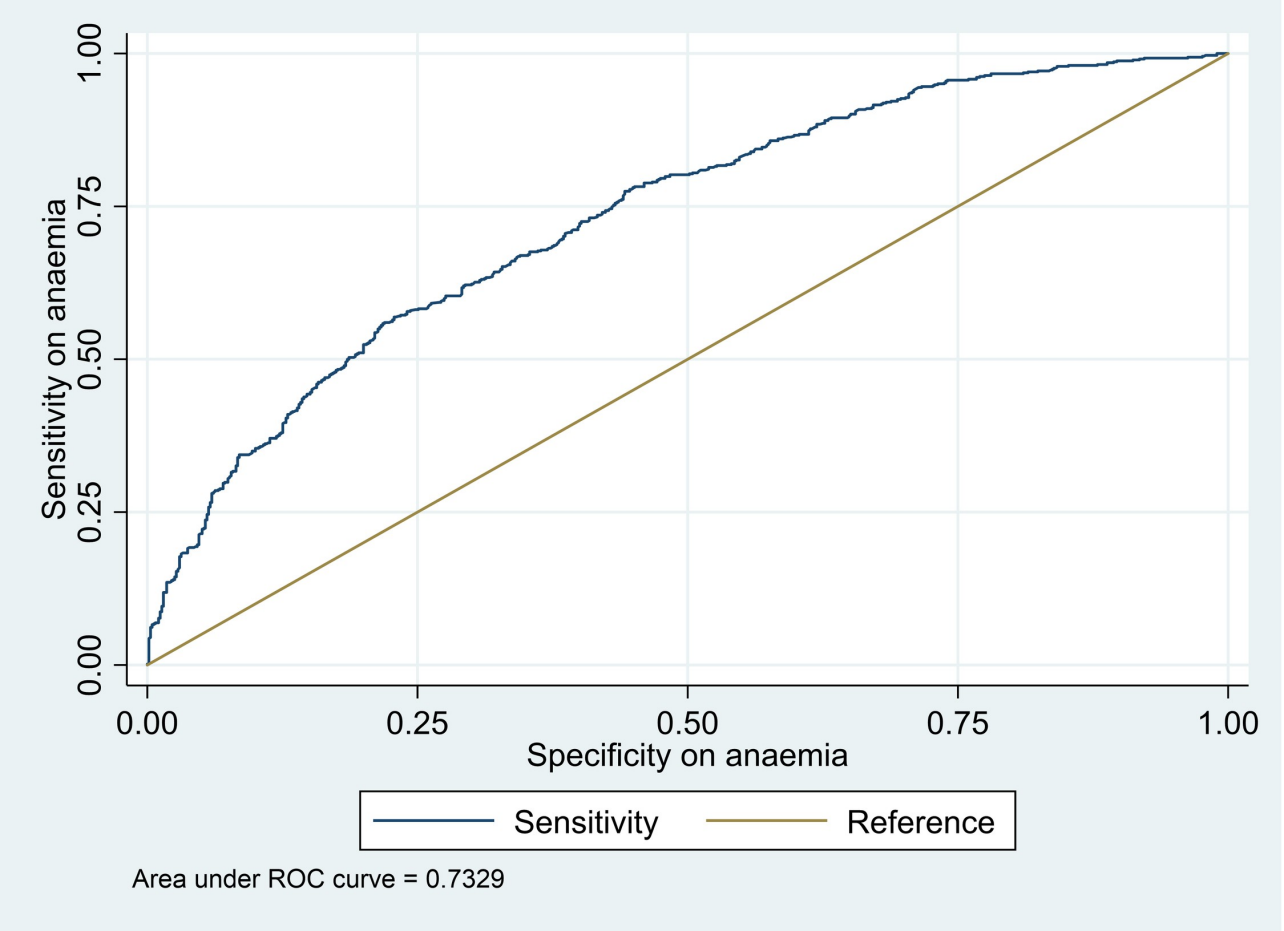
Anaemia logistic regression model showing sensitivity and specificity to factors associated anaemia in a receiver operating characteristics (ROC) curve.

The anaemia model was able to predict correctly about various factors in association with anaemia by 73% depicted as area under the receiver operating characteristic (ROC) curve, developed following the logistic regression (Fig 5).

### Cognitive and psychomotor functions

Boys had significantly higher VO2max compared to girls (adjusted coefficient of 3.7, 95%CI 2.7–4.8, P<0.001). Stunted children had lower VO2max compared to normal children (adjusted coefficients of -1.9, 95% CI -3.0- -0.8, P<0.01). Malaria and anaemia reduced VO2max but the correlation was not statistically significant. Schistosomiasis significantly correlated with reduction of VO2max at unadjusted coefficient of -2.63 (95%CI -4.9–0.3, *p = 0*.*02*), but could not have an effect on adjustment with other factors. All other factors did not show significant correlation to VO2max. Boys had high cognitive scores compared to girls (adjusted coefficient of 3.7 (95%CI 0.4–7.0, *p = 0*.*03*). Malaria and anaemia did not have significant correlation with cognitive scores. History of confirmed malaria the previous one month was associated with reduction in cognitive scores at adjusted coefficient of -4.1 (95% CI -7.7–0.6, *p = 0*.*02*). Schools were included in the model to control any school specific confounders, their respective correlation to psychomotor or cognitive function model are as shown on Table 6. The correlation between factors assessed on the model to either psychomotor or cognitive function, did not change on robust standard error estimation and even when bootstrap technique was applied to the models.

**Table 6:** Univariate and multivariate analysis of factors influencing psychomotor and cognitive function among primary schoolchildren in Muheza, northestern Tanzania.

		Overall		Univariate analysis			Multivariate analysis		
Variables		n	Mean score (SD)	Unadjusted coefficients (95%CI)		P-value	Adjusted coefficients (95% CI)		P-value
***Psychomotor function model using VO2max***									
Sex	Male	164	50.3 (4.9)	3.87	(2.77–4.97)	<0.001	3.72	(2.65–4.78)	<0.001
Stunting (HAZ<-2zscore)		127	47.4 (5.5)	-1.93	(-3.11–0.75)	0.001	-1.93	(-3.04–0.82)	0.001
History of malaria last month		98	47.9 (5.4)	-0.96	(-2.22–0.29)	0.13			
Malaria	Positive	88	48.3 (4.7)	-0.43	(-1.76–0.90)	0.53			
Anaemia	Yes	156	48.1 (5.1)	-0.78	(-1.96–0.40)	0.19			
*S*. *haematobium* infestation		22	46.1 (5.0)	-2.63	(-4.91–0.35)	0.02			
School*									
	Kwakibuyu	40	46.1 (4.5)						
	Bwitini	50	49.3 (4.5)	3.25	(1.12–5.38)	0.003	2.16	(0.17–4.14)	0.03
	Mkulumilo	50	49.6 (4.6)	3.56	(1.43–5.69)	0.001	2.8	(0.83–4.78)	0.01
	Pangamlima	50	48.1 (5.2)	2.01	(-0.12–4.13)	0.07	1.23	(-0.78–3.24)	0.23
	Songa Kibaoni	46	46.8 (5.9)	0.73	(-1.44–2.90)	0.510	0.34	(-1.66–2.35)	0.74
	Mhamba	36	49.6 (5.3)	3.49	(1.18–5.79)	0.003	2.47	(0.31–4.63)	0.03
	Heinkele	34	50.2 (5.8)	4.16	(1.82–6.50)	0.001	3.51	(1.35–5.67)	0.002
***Cognitive function model using cognitive score***									
Sex	Male	162	16.9 (15.5)	2.84	(-0.55–6.23)	0.1	3.77	(0.49–7.05)	0.02
History of malaria last month		96	12.5 (13.9)	-4.52	(-8.14- -0.90)	0.02	-4.12	(-7.65–0.58)	0.02
Malaria	Positive	87	17.7 (15.5)	2.97	(-0.84–6.77)	0.13	2.88	(-0.76–6.53)	0.12
Anaemia	Yes	155	14.8 (14.8)	-1.56	(-4.95–1.84)	0.37			
*S*. *haematobium* infestation		22	19.9 (15.4)	4.56	(-1.98–11.11)	0.17			
School*									
	Kwakibuyu	40	16.3 (15.6)						
	Bwitini	50	10.8 (11.7)	-5.45	(-11.34–0.44)	0.07	-6.99	(-13.09–0.89)	0.03
	Mkulumilo	50	10.4 (13.1)	-5.87	(-11.76–0.02)	0.05	-7.06	(-13.03–1.08)	0.02
	Pangamlima	49	14.2 (14.4)	-2.05	(-7.96–3.87)	0.50	-2.75	(-8.79–3.30)	0.37
	Songa Kibaoni	45	27.3 (14.0)	11.04	(5.01–17.07)	<0.001	11.34	(5.08–17.60)	<0.001
	Mhamba	36	17.8 (16.4)	1.58	(-4.79–7.96)	0.63	0.96	(-5.50–7.41)	0.77
	Heinkele	33	13.3 (13.9)	-2.98	(-9.50–3.55)	0.37	-3.15	(-9.89–3.58)	0.36

*school arranged in ascending order following mean location altitude.

## Discussion

In this study, we found asymptomatic malaria prevalence at 26%, stunting 21% and anaemia 49%, while the prevalence of antimalarial drug resistance markers were a mixture of haplotypes regarding PfMDR1; e.g. PfMDR1-NFD (52%) and NYD (47%), and close to wild type fixation for PfCRT-CVMNK (99%) and PfExo- E (100%). Factors such as; age group (10–15 years), not sleeping under a bednet, low socioeconomic status, parents’ or guardian with low level of education, more number of children in a household, and less number of rooms in a household significantly attributed to higher malaria infection in the study area. Even though, anaemia has multiple causalities, in this study, asymptomatic malaria infection attributed significantly to 61% of anaemia, followed by stunting that attributed to 34% of anaemia among the study participants with malaria and stunting, respectively.

Despite geographical variations across communities, schoolchildren shared some parameters for risk of infection where, children from households located at low altitude had more malaria infection than those in high altitude areas, this has also been reported in other studies [51,52,54]. However, the significance disappeared when analysed in a multivariate malaria model. Other factors observed include, children whose parents or guardian had low (primary) level or no education were at high odds of getting malaria infection (OR = 2.2 and 2.3, respectively). This attributed to 45% and 7%, respectively of malaria infection in the population. Of note, the proportion of parents or guardian with no education was low (13%) with vast majority being those with primary education (81%). This explains the population attributable fraction difference of the two educational levels in terms of risks. Generally, comparing population with low or non-educated to those with secondary or above education level, there is a significantly high attribution to malaria infection on the low or non-educated group. This is consistent with findings from other studies that reported children from mothers whose education level was beyond primary school were less likely to be malaria-positive [55–57]. Overcrowding was another risk that increased odds of malaria infection. Children from households with large number of children in the household were at high risk of malaria infection, this was also consistent with other studies that reported increasing household size was positively correlated with increased risk of infection [55]. Overcrowding and deficient housing is in most cases a product of poverty. Low socioeconomic status has shown to attribute to about 11% of malaria infection in the study population. This has been accompanied with high number of children (overcrowding) and low number of rooms in a household which both attributed to 13% and 12%, respectively of malaria infection in the population, also noted elsewhere [58]. In the past, the low socioeconomic status had contributed vastly to high malaria infection rates in the endemic communities of Africa leading to inclusion of malaria in a group of diseases of poverty [59].

Preventive programs such as bednets and increased literacy level in the communities have attributed significantly to reduced malaria infection rates in the communities [60,61]. In our study, not sleeping under a bednet attributed to 34% of malaria infection and 40% of anaemia among children not using bednets, and attributed to 8% of malaria infection and 9% of anaemia in the study population. However, use of bednets did not provide full protection either. 24% and 47% of those who used bednets have had malaria infection and anaemia, respectively. Though some studies have reported low bednet usage among schoolchildren [62], we cannot ignore the partial effectiveness of bednets in the study area, where over 80% of study participants reported their nets to have holes i.e. torn out. Another explanation could be due to overcrowding, where a bednet is insufficient in covering all occupants of the shared bed, especially in a limited sleeping space. Thus, a bednet replacement or school based distribution programme could be ideal. Nonetheless, it has also been reported that most of bednets used in Africa including Tanzania, have been impregnated with pyrethroid insecticides which are currently doomed to failure due to persistent resistance of mosquitoes to pyrethroids [60,63].

The shifting trend of asymptomatic malaria infection with increased risk from low age to young adolescents is obvious; where children aged 10 to 15 years had 1.4 (*p = 0*.*03*) adjusted odds of malaria infection compared to children aged 5–9 years old. In addition, this age group (10–15 years) had an attributable fraction to malaria infection of 28%, and had a population attributable fraction of 12%. A Tanzanian national wide school malaria survey conducted in 2014 and 2019 showed a similar trend as the present study [11,64] and so did the Malawian study [12]. This shift was not observed in similar studies conducted from Uganda [53] and Kenya [47] where children aged 6–10 years had higher malaria prevalence compared to those aged 11–14 years. It is worth noting that in these studies, the age cut-off point at 10 years may have played a role, given the median age (9 years) in our study population. It may also be the long time span between the Ugandan or Kenyan study and our study (almost 10 years), that could be subjected to the epidemiological trend accompanying age shift [13,65–68]. The shift in malaria epidemiology has been attributed to increased interventions that formerly left out schoolchildren and some included children aged under ten years [65,66,69]. Nonetheless, several studies have called for malaria interventions in school-aged children or young adolescents [12,53,70], signifying the public health importance of malaria infection in schoolchildren. In addition, given the ongoing shift in prevalence of malaria to children older than 9 years, the current classification of malaria endemicity that is widely used on malaria atlas and by the national malaria control programmes [66], using the annual mean prevalence of *P*. *falciparum* infection among children aged 2–10 years (*PfR*_*2-10*_) cannot be precise when dealing with schoolchildren. When planning malaria control programmes implementation, the decision based on *PfR*_*2-10*._, should be improved by a *PfR*_*10-15*_ and or a *PfR*_*5-15*_.

We also explored the prevalence of antimalarial drug resistance in schoolchildren. This gave us more information on malaria infection and management in the study community thereof. We saw high sensitivity to Amodiaquine (AQ) and Chloroquine (CQ) (*Pfcrt*-CVMNK haplotype at 99%). There was also resistance to *Pfmdr*1-NFD haplotype at 47%, normally associated with resistance to Lumefantrine (a partner drug in Artemether Lumefantrine-ALU) the first line antimalarial treatment drug in Tanzania [71,72]. The increased sensitivity to AQ and CQ could be due to the fact that AQ and CQ were used in Tanzania and then stopped in 2000s, and so have regained their sensitivity as described elsewhere [73]. We did not test drug clearance rates in the population. However, given the prevalence of resistant genes to the first line antimalarial drug could possibly mean the drug (ALU) has undergone or is undergoing a resistant track, worth monitoring by the NMCP. In practice, some parents / guardians may not let their children complete the treatment scheme (ALU), i.e. to keep it for future clinical episodes for this or any other child. This practice, if happening, propagates resistance. The substantial high prevalence of *Pfdhfr* and *Pfdhps* sulphadoxine pyrimethamine (SP) drug resistance, was expected as it was earlier documented [73]. This could hinder effectiveness of the intermittent preventive treatment of malaria in pregnant women (IPTp), where SP still is being considered as the drug of choice. In this regard, before implementing any IPT programme tackling malaria in school children (IPTsc) as recommended recently [23], markers of drug resistance levels should be explored upfront in order to define and pick the most appropriate molecules.

Children with malaria have been at high odds of having anaemia, also noted in other studies [11,43,47]. This could be due to a direct effect of malaria parasite on haemolysis and decreased red blood cell production [74,75]. Since malaria attributed to 61% of anaemia in children having malaria, and 16% of anaemia in the study population, there is a high need to control malaria. In addition, children whose parents or guardians were illiterate attributed to 43% of anaemia in this group, and a 5% attribution to anaemia in the population. Since parents’ or guardians’ literacy also plays a role on malaria, then it is worth intervening. The importance of having high literacy rate in the society was observed in other studies as well [43,47]. It is expected that a literate parent or guardian either to at least know the basics of disease prevention, having studied either at schools or read and understood malaria preventive and nutrition educational massages from the media. The educational messages are vital for successful implementation of school health programmes as they increase parents /guardian’s awareness, cooperation and adherence to established interventional programmes such as for malaria and nutrition [76]. The presence of school food programme that normally involve parents and or guardians contributions, was associated with 50% reduction of anaemia in a Kenyan study [47]

We cannot ignore other factors related to anaemia, such as nutrition (stunting), STH and *S*. *haematobium* infestations [47,77]. A study in Tanzania reported more than half of school children had nothing to eat before going to school, and over 70% had nothing to eat while in school [78]. Also a national school malaria and nutrition survey(SMNS) conducted in 2019 reported the mean individual dietary scores (IDDS) and households (HDDS) were both low [64]. Generally school feeding programme has been slowly adopted and with less sustainability, especially in the low income countries [79]. STH and schistosomiasis are well known to relate to anaemia [7,19,77], however, they did not have significant contribution to anaemia in the study population. In our study, STH was less prevalent, but at a school (Kwakibuyu) that had 75% anaemia prevalence, it also had high prevalence of STH and *S*. *haematobium* infestation, 3% and 30%, respectively. School deworming programmes are practiced yearly in Tanzania [20,21,80], but it seems there are still some pockets especially in hard to reach areas such as Kwakibuyu village where coverage could be sub optimal (55% of children reported to have received deworming drugs including those for schistosomiasis in the past 6 months). It is also possible that drugs such as Praziquantel used for treatment and control of schistosomiasis wanes too early that only a single round per year would not be sufficient for protection in the entire period [81], especially in areas with high transmission like Kwakibuyu.

Malaria related anaemia has been associated with impaired cognitive and psychomotor function as documented elsewhere [6,23,39,53,82,83]. However, in our study, neither malaria nor anaemia had any significant impact on cognitive or psychomotor function. This could be due to the fact that malaria parasite densities observed was not high enough (median of 672 asexual stages/μL) to interfere the cognitive function as was noted in Uganda [43] where a dose–response relationship between parasite density and sustained attention was observed. Nevertheless, a similar study done in Kenya [47] showed no observed association between malaria infection and cognitive function. This could be explained by geographic locations, where the Kenyan study and our study site both share similar altitudes. i.e. being near the coast and hence possibly have similar underlying malaria transmission intensity and prevalence compared to Uganda [43]. In addition, since we observed high odds of malaria infection in children aged 10–15 years, the children we selected (class 4 and 5) shared a lot in common for levels of asymptomatic parasitaemia and probably have biologically acclimatised to their health status quo. This kind of children selection was not done in Uganda.

On the other hand, there was a significant association of low cognitive scores attributed to history of malaria infection in the past one month. This could be confirming the narrative that cognitive function derangement is an outcome of cumulative malaria infection as noted in Kenya [47] and since this was a cross sectional design it falls short of capturing chronic infection over time [43]. The other plausible reason could be, we used a subset of students of class 4 and 5 who would generally understand instructions well enough that one can still score high for the sustained attention test that runs approximately 15–30 minutes, despite having underlying parasitaemia or even with mild malaria symptoms (we observed 10 children scoring better despite having fever). In addition, stunting was significantly associated with reduction in VO2max while male sex increased both VO2max and cognitive scores. This could be due to nutritional effect over time on anaemia, and that, boys are known for their physical activities such as regular football game making them fit under normal circumstances.

Our study findings cannot escape the limitations of a cross sectional design especially on issues related to causality [84,85]. Yet still, the risks and their attribution to the outcome malaria, anaemia and drug resistance cannot be reversed in terms of explaining causality. With the fact that we included several factors in the final analysis models, we are pretty sure that the models controlled for any potential confounders, as was done in similar studies [43,47]. Though we did not assess nutritional dietary measures uptake as one of risk factors for anaemia, we still think calculations of standard nutritional status (BMI, HAZ, and WAZ) gave a meaningful picture. Therefore, we think, the attribution we got is quite robust to explain the effect. In addition, asymptomatic parasitaemia are generally low density and reliance on microscopy for identification is dependent on the technicians and intensity of infection. However, we used well-trained expert microscopists with massive experience in the field, and that microscopy is the gold standard for malaria test. Of course getting to the level of sub microscopic parasitaemia could have provided some further important details on a probable high human parasite malaria reservoir population (i.e. school children) [12,86]. The significant differences in baseline characteristics between clinically evaluated and those not clinically evaluated, cannot be regarded as study limitation as they followed exclusion criteria as per the main study protocol. This analysis mainly focused on clinically evaluated schoolchildren alone. In addition, the minor difference shown in participants to non-participants on the supplementary table (S1 Table) could be by chance. Another explanation is that, the non-participants group comprised of a larger portion of children under-five (who were not registered at schools). They were termed as of school-age by parents/ guardians either by mistake or out of their eagerness to let their children join the main study due to the perceived benefit from care provided in the trial [23,87]. Therefore this could not warrant for a selection bias, as the difference are typical of those seen in under-fives when compared to 5–15 years old (S2 Table).

Studies [10,43,47,88] have shown somehow similar risks for malaria infection and anaemia in school children, however, they did not quantify the attribution of such risks to infection and to the general population. Our study therefore, has detailed on risk and quantified their attribution to the population, making it easier for priority setting when dealing with school health. Malaria, illiteracy and stunting in comparison to other risks have been so obvious on their contribution to anaemia. Interventional packages for malaria, increased literacy and nutritional deficiencies are highly needed in the study area. Cohee *et al*., 2021 have summarised quite a number of interventions addressing malaria in schoolchildren, and proposed increasing access to testing and treatment in schools or providing chemoprevention to school-age children as possible approaches [89]. We give high prospects to chemoprevention (mainly IPTsc) given the effectiveness, practicability and feasibility of such intervention [23] compared to testing and treating methods. This is due to the fact that most infections in school children are asymptomatic, that may also be sub microscopic, and that the current testing kits are limited to varying sensitivities [48,90–94], and such a method would not be pragmatically feasible. We also suggest malaria preventive measures should go hand in hand with tackling nutrition (stunting) and a comprehensive interventional-based health education in schools and communities at large.

## Conclusion

This study has highlighted the extent of attribution of each risk identified to malaria and anaemia. We have shown that malaria, illiteracy and stunting play a key role on anaemia, which would in turn, contribute to cognitive impairment. Current malaria control tools are suboptimal, especially for malaria control in school-aged children. Given malaria infection in schoolchildren is mostly asymptomatic; an addition of intervention programmes such as IPTsc would probably give potential solution while calling for improvement on the current tools such as bednet use and school food programme, community based (customised) health education with emphasis to nutrition and malaria control.

## Supporting information

S1 Table Description of eligible children and their households surveyed in the study comparing characters for participants and non-participants. (DOCX)

S2 Table Description of eligible children and their households surveyed in the study comparing characters for age 5–15 years and under fives. (DOCX)

S3 Table Different markers of antimalarial drug resistance assessed from schoolchildren in Muheza, Tanzania. (DOCX)

S4 Table *Pf K13* mutations in propeller region. (DOCX)
