# Correction: Attributable risk factors for asymptomatic malaria and anaemia and their association with cognitive and psychomotor functions in schoolchildren of north-eastern Tanzania

**DOI:** 10.1371/journal.pone.0318828

**Published:** 2025-01-30

**Authors:** The PLOS ONE Staff

In Table 4, the column headings were incorrectly formatted. Please see the corrected Table 4 here.

**Table 4:** Univariate and multivariate analysis of factors associated with asymptomatic malaria infection among primary schoolchildren in Muheza, north-eastern Tanzania.

			n/N with *Plasmodium* infection	Univariate analysis			Multivariate analysis				
Variables				Crude odds ratio (95% CI)		P-value	Adjusted odds ratio (95% CI)		P-value	AF	PAF
Sex		Female	159/692								
		Male	229/779	1.40	(1.10–1.77)	0.01	1.27	(0.97–1.66)	0.08		
Age group		5–9 years	182/808								
		10–15 years	206/663	1.55	(1.23–1.96)	<0.001	1.38	(1.04–1.84)	0.03	0.28	0.12
Nutritional status											
	WAZ <-2zscore		121/416	1.21	(0.94–1.56)	0.14					
	HAZ <-2 zscore		110/303	1.82	(1.39–2.39)	<0.001	1.15	(0.82–1.62)	0.41		
	BMI <-2zscore		102/427	0.83	(0.64–1.08)	0.17					
Anaemia			256/732	2.47	(1.94–3.14)	<0.001	2.56	(1.91–3.42)	<0.001		
STH infestation			5/16	1.27	(0.44–3.68)	0.66					
*S*. *haematobium* infestation			41/122	1.46	(0.98–2.17)	0.06	1.39	(0.85–2.25)	0.19		
Number of children in a HH			363/1354	1.15	(1.06–1.25)	<0.001	1.17	(1.07–1.28)	<0.001	0.15	0.13
Few rooms in a HH			371/1396	1.13	(1.03–1.24)	0.01	1.12	(1.01–1.25)	0.04	0.11	0.10
Household Altitude			363/1007	0.99	(0.99–1.00)	0.001	1.00	(1.00–1.00)	0.57		
SES	Higher SES		90/464								
	Moderate SES		136/479	1.65	(1.22–2.23)	<0.001	1.60	(1.13–2.26)	0.01	0.38	0.12
	Low SES		162/528	1.84	(1.37–2.47)	<0.001	1.43	(1.12–2.07)	0.047	0.30	0.11
Head of household education level											
Secondary or above			12/86								
		Primary	307/1141	2.27	(1.22–4.24)	0.01	2.22	(1.09–4.52)	0.03	0.55	0.45
None			56/178	2.83	(1.42–5.63)	0.001	2.30	(1.04–5.07)	0.04	0.57	0.07
Eaves open			302/1128	1.03	(0.75–1.41)	0.83					
Not sleeping under a bednet			116/318	1.86	(1.42–2.43)	<0.001	1.52	(1.12–2.07)	0.01	0.34	0.07
School*		Heinkele	51/269								
		Mhamba	46/228	1.08	(0.69–1.69)	0.73	0.96	(0.57–1.60)	0.87		
		Songa Kibaoni	53/237	1.23	(0.80–1.90)	0.35	1.55	(0.93–2.59)	0.09		
		Bwitini	52/220	1.32	(0.86–2.05)	0.21	1.45	(0.80–2.65)	0.22		
		Mkulumilo	63/190	2.12	(1.38–3.26)	0.001	2.03	(1.15–3.64)	0.02	0.51	0.07
		Kwakibuyu	59/167	2.34	(1.50–3.63)	0.001	2.03	(1.05–3.95)	0.04	0.51	0.06
		Pangamlima	64/160	2.85	(1.84–4.42)	0.001	3.80	(2.17–6.65)	<0.001	0.74	0.08

*school arranged in ascending order following malaria prevalence, AF = Attribution fraction = (aOR-1) /aOR, PAF = Population attribution fraction = [AF*Proportion of a factor in a population]; where aOR is adjusted odds ratio. SES = Socioeconomic status; Factors used in the adjusted model include anaemia, sex, age-group, socioeconomic status, stunting (zhaz), sleep under a bednet, education of head of household, schistosomiasis infestation, location altitude, number of children in a household, number of rooms in a household, and school.

The publisher apologizes for the errors.
