# Supplementary material for: Attributable risk factors for asymptomatic malaria and anaemia and their association with cognitive and psychomotor functions in schoolchildren of north-eastern Tanzania
Source: PLoS One. 2022 May 26;17(5):e0268654. doi: 10.1371/journal.pone.0268654 (PMC9135275; doi:10.1371/journal.pone.0268654)
Supplement: S1 Table — (DOCX) [file pone.0268654.s001.docx]

**S1 Table 1 Description of eligible children and their households surveyed in the study comparing characters for participants and non-participants**

| **Variable** | **Category** | **All (N=1797)** | **Comparison** | | **p-value** |
| --- | --- | --- | --- | --- | --- |
|  |  | **mean or %** | **Participants (N=1587)** | **Non-Participants (N=210)** |  |
| Mean age (SD) | all, mean (SD) | 9.1 (2.6) | 9.1 (2.6) | 8.6 (3.0) | **0.01** |
| Sex | Male, n (%) | 961 (53.5) | 837 (52.7) | 124 (59.1) | 0.09 |
|  | Female, n (%) | 836 (46.5) | 750 (47.3) | 86 (41.0) |  |
| Had malaria last month | n (%) | 740 (41.2) | 669 (42.2) | 71 (33.8) | **0.02** |
| Malaria tested last month (confirmed), n (%) | | 555 (75.5) | 504 (75.9) | 51 (71.8) | 0.45 |
| Common malaria treatment place | Health facility, n (%) | 437 (71.1) | 375 (70.0) | 62 (78.5) | 0.12 |
|  | Drug shop, n (%) | 171 (27.8) | 154 (28.7) | 17 (21.5) | 0.18 |
|  | Traditional, n (%) | 7 (1.1) | 7 (1.3) | 0 (0) | 0.31 |
| Slept under a bednet last night, Yes % |  | 1426 (79.4) | 1252 (78.9) | 174 (82.9) | 0.18 |
| Net is LLIN, n (%) |  | 1281 (89.8) | 1130 (90.3) | 151 (86.8) | 0.15 |
| Net with holes, n (%) |  | 1182 (82.9) | 1037 (82.8) | 145 (83.3) | 0.87 |
| Received anthelminthic medication last 6 months, n (%) | | 974 (54.2) | 876 (55.2) | 98 (46.7) | **0.02** |
| Household location altitude, Mean(SD) -meters | | 224.6 (34.4) | 223.7 (34.7) | 231.4 (31.2) | **0.00** |
| Household socioeconomic status, Low, n (%) | | 914 (50.9) | 814 (51.3) | 100 (47.6) | 0.32 |
|  | Secondary or high, n (%) | 213 (12.3) | 189 (12.4) | 24 (11.7) | 0.77 |
| Parent's level of education | Primary, n (%) | 1412 (81.8) | 1239 (81.5) | 173 (84.4) | 0.31 |
|  | None, n (%) | 101 (5.9) | 93 (6.1) | 8 (3.9) | 0.21 |
| Houses with eaves open, n (%) |  | 1367 (81.0) | 1210 (81.3) | 157 (78.9) | 0.41 |
| Livestock live inside house, n (%) |  | 939 (75.8) | 829 (75.2) | 110 (80.1) | 0.14 |
| Number of people in a HH, Mean (SD) | | 6.0 (2.1) | 5.9 (2.1) | 6.2 (2.2) | 0.12 |
| Number of children in a HH, Mean (SD) | | 3.4 (1.5) | 3.3 (1.5) | 3.5 (1.5) | 0.11 |
| Number of rooms in HH, Mean (SD) | | 3.4 (1.3) | 3.4 (1.3) | 3.4 (1.2) | 0.41 |
| Number of rooms for sleeping, Mean (SD) | | 2.3 (0.9) | 2.3 (0.9) | 2.5 (0.9) | **0.01** |

*Legend: HH=House hold, LLIN= Long lasting Insecticide treated net, STH=Soil Transmitted Helminths, SD=Standard Deviation*
