# Supplementary material for: Attributable risk factors for asymptomatic malaria and anaemia and their association with cognitive and psychomotor functions in schoolchildren of north-eastern Tanzania
Source: PLoS One. 2022 May 26;17(5):e0268654. doi: 10.1371/journal.pone.0268654 (PMC9135275; doi:10.1371/journal.pone.0268654)
Supplement: S2 Table — (DOCX) [file pone.0268654.s002.docx]

**S1 Table 2 Description of eligible children and their households surveyed in the study comparing characters for age 5-15 years and under fives**

| **Variable** | **Category** | **All (N=2,628)** | **Comparison** | | **p-value** |
| --- | --- | --- | --- | --- | --- |
|  |  | **mean or %** | **Age 5-15 years (N=2,109)** | **Age under 5 years (N=519)** |  |
| Mean age (SD) | all, mean (SD) | 8.0 (4.0) | 9.5 (2.9) | 2.2 (1.3) | **<0.01** |
| Sex | Male, n (%) | 1377 (52.4) | 1114 (52.8) | 263 (50.7) | 0.38 |
|  | Female, n (%) | 1251 (47.6) | 995 (47.2) | 256 (49.3) |  |
| Had malaria last month | n (%) | 1003 (38.2) | 842 (39.9) | 161 (31.0) | **<0.01** |
| Malaria tested last month (confirmed), n (%) | | 759 (76.1) | 629 (75.2) | 130 (80.8) | 0.13 |
| Common malaria treatment place | Health facility, n (%) | 638 (70.3) | 522 (71.5) | 116 (65.2) | 0.10 |
|  | Drug shop, n (%) | 260 (28.6) | 200 (27.4) | 60 (33.7) | 0.10 |
|  | Traditional, n (%) | 10 (1.1) | 8 (1.1) | 2 (1.1) | 0.97 |
| Slept under a bednet last night, Yes % | | 2135 (81.2) | 1671 (79.2) | 464 (89.4) | **<0.01** |
| Net is LLIN, n (%) |  | 1907 (89.3) | 1499 (89.7) | 408 (87.9) | 0.27 |
| Net with holes, n (%) |  | 1738 (81.4) | 1394 (83.4) | 344 (74.1) | **<0.01** |
| Received anthelminthic medication last 6 months, n (%) | | 1182 (45.0) | 1118 (53.0) | 64 (12.3) | **<0.01** |
| Household location altitude, Mean(SD) -meters | | 225.6 (34.9) | 225.0 (34.7) | 228.0 (35.3) | 0.09 |
| Household socioeconomic status, Low, n (%) | | 1314 (50.0) | 1052 (49.9) | 262 (50.5) | 0.81 |
|  | Secondary or high, n (%) | 298 (11.8) | 242 (11.9) | 57 (11.3) | 0.71 |
| Parent's level of education | Primary, n (%) | 2072 (81.7) | 1664 (81.9) | 408 (80.6) | 0.50 |
|  | None, n (%) | 167 (6.6) | 126 (6.2) | 41 (8.1) | 0.12 |
| Houses with eaves open, n (%) |  | 2013 (81.4) | 1604 (81.1) | 409 (82.5) | 0.48 |
| Livestock live inside house, n (%) |  | 1385 (75.6) | 1120 (76.1) | 265 (73.2) | 0.25 |
| Number of people in a HH, Mean (SD) | | 6.1 (2.2) | 6.0 (2.2) | 6.5 (2.3) | **<0.01** |
| Number of children in a HH, Mean (SD) | | 3.5 (1.5) | 3.4 (1.5) | 3.7 (1.5) | **<0.01** |
| Number of rooms in HH, Mean (SD) | | 3.4 (1.3) | 3.4 (1.3) | 3.3 (1.3) | 0.11 |
| Number of rooms for sleeping, Mean (SD) | | 2.3 (1.0) | 2.3 (1.0) | 2.2 (1.0) | 0.06 |

*Legend: HH=House hold, LLIN= Long lasting Insecticide treated net, STH=Soil Transmitted Helminths, SD=Standard Deviation*
