# Supplementary material for: Attributable risk factors for asymptomatic malaria and anaemia and their association with cognitive and psychomotor functions in schoolchildren of north-eastern Tanzania
Source: PLoS One. 2022 May 26;17(5):e0268654. doi: 10.1371/journal.pone.0268654 (PMC9135275; doi:10.1371/journal.pone.0268654)
Supplement: S3 Table — (DOCX) [file pone.0268654.s003.docx]

**S1 Table 3 Different markers of antimalarial drug resistance assessed from schoolchildren in Muheza, Tanzania**.

| ***Pfdhfr*** | | | ***Pfdhps*** | | | ***Pfexo*** | | | ***Pfmdr1*** | | | ***Pfcrt*** | | |
| --- | --- | --- | --- | --- | --- | --- | --- | --- | --- | --- | --- | --- | --- | --- |
| **Codon** | **Genotype** | **n (%)** | **Codon** | **Genotype** | **n (%)** | **Codon** | **Genotype** | **n (%)** | **Codon** | **Genotype** | **n (%)** | **Codon** | **Genotype** | **n (%)** |
| **51** | **N** | 0 (0) | **431** | **I** | 216 (100) | **415** | **E** | 198 (100) | **86** | **N** | 213 (97.3) | **72** | **S** | 0 (0) |
|  | **I** | 245 (97.6) |  | **V** | 0 (0) |  | **G** | 0 (0) |  | **Y** | 1 (0.5) |  | **C** | 222 (100) |
|  | **NI** | 6 (2.4) |  | **IV** | 0 (0) |  | **EG** | 0 (0) |  | **F** | 0 (0) |  | **SC** | 0 (0) |
|  |  |  | **436** | **A** | 0 (0) |  |  |  |  | **NF** | 0 (0) | **73** | **V** | 222 (100) |
| **59** | **C** | 7 (2.8) |  | **S** | 193 (100) |  |  |  |  | **NY** | 5 (2.3) | **74** | **I** | 2 (0.9) |
|  | **R** | 234 (92.9) |  | **SA** | 0 (0) |  |  |  | **184** | **Y** | 104 (48.19 |  | **M** | 222 (99.1) |
|  | **CR** | 11 (4.3) |  | **F** | 0 (0) |  |  |  |  | **F** | 81 (37.5) |  | **IM** | 0 (0) |
|  |  |  | **437** | **A** | 7 (3.7) |  |  |  |  | **YF** | 31 (14.4) | **75** | **N** | 220 (99.1) |
| **108** | **N** | 258 (100) |  | **G** | 182 (95.3) |  |  |  | **1034** | **S** | 211 (100) |  | **E** | 2 (0.9) |
|  | **S** | 0 (0) |  | **GA** | 2 (1.0) |  |  |  |  | **C** | 0 (0) |  | **NE** | 0 (0) |
|  | **NS** | 0 (0) | **540** | **K** | 5 (2.5) |  |  |  |  | **SC** | 0 (0) | **76** | **K** | 219 (99.1) |
|  |  |  |  | **E** | 188 (95.4) |  |  |  | **1042** | **N** | 211 (100) |  | **T** | 2 (0.9) |
| **164** | **I** | 253 (199) |  | **KE** | 4 (2.0) |  |  |  |  | **D** | 0 (0) |  | **KT** | 0 (0) |
|  | **L** | 0 (0) | **581** | **G** | 63 (35.6) |  |  |  |  | **ND** | 0 (0) |  |  |  |
|  | **IL** | 0 (0) |  | **A** | 114 (64.4) |  |  |  | **1246** | **D** | 206 (98.1) |  |  |  |
|  |  |  |  | **AG** | 0 (0) |  |  |  |  | **Y** | 4 (1.9) |  |  |  |
|  |  |  | **613** | **A** | 217 (100) |  |  |  |  | **DY** | 0 (0) |  |  |  |
|  |  |  |  | **S** | 0 (0) |  |  |  |  |  |  |  |  |  |
|  |  |  |  | **SA** | 0 (0) |  |  |  |  |  |  |  |  |  |
