# Supplementary material for: Attributable risk factors for asymptomatic malaria and anaemia and their association with cognitive and psychomotor functions in schoolchildren of north-eastern Tanzania
Source: PLoS One. 2022 May 26;17(5):e0268654. doi: 10.1371/journal.pone.0268654 (PMC9135275; doi:10.1371/journal.pone.0268654)
Supplement: S4 Table — (DOCX) [file pone.0268654.s004.docx]

**S1 Table 4 *Pf K13* mutations in propeller region**

| **Nucleotide position** | **Reference *Pf3d7*** | **Genotype** |
| --- | --- | --- |
| **1359** | T | TC |
| **1407** | C | T |
| **1486** | G | AG |
| **1509** | G | AG |
| **1699** | G | AG |
| **1738** | T | TC |
| **1765** | G | AG |
| **1913** | G | AG |
| **1923** | T | TC |
| **1996** | G | C |
| **1996** | G | GC |
